# Variations in NAG-1 expression of human gastric carcinoma and normal gastric tissues

**DOI:** 10.3892/etm.2013.1361

**Published:** 2013-10-24

**Authors:** GONGLI YANG, QINHUA TAN, YONGMEI XIE, BIN WEI, ZHIXIN CHEN, CHENGWEI TANG, SHENGBAO LI, CHUNHUI WANG

**Affiliations:** 1 Department of Gastroenterology, Nanfang Hospital, Southern Medical University, Guangzhou, Guangdong 510515, P.R. China; 2 Department of Gastroenterology, West China Hospital, Sichuan University, Chengdu, Sichuan 610041, P.R. China; 3 Department of Gastroenterology, Taihe Hospital, Hubei Medical University, Shiyan, Hubei 442000, P.R. China

**Keywords:** gastric carcinoma, nonsteroidal anti-inflammatory drug activated gene-1

## Abstract

Nonsteroidal anti-inflammatory drug-activated gene-1 (NAG-1), a member of the transforming growth factor β (TGF-β) superfamily, has been demonstrated to possess antitumorigenic and proapoptotic activities in gastric cancer cells. In the present study, the expression of NAG-1 was assessed in human gastric carcinoma, tumor-adjacent normal tissues and normal gastric mucosa, with the aim to investigate the role of NAG-1 in the carcinogenesis and development of gastric carcinoma. NAG-1 protein expression was evaluated using immunohistochemical staining, while the expression of NAG-1 mRNA was evaluated using reverse transcription-polymerase chain reaction. It was observed that adenocarcinoma tissues had a lower expression of NAG-1 than normal gastric tissues. Furthermore, moderately and well-differentiated adenocarcinoma tissues expressed more NAG-1 protein than the poorly differentiated adenocarcinoma tissues. The expression of NAG-1 protein in adenocarcinoma tissues did not correlate with tumor-node-metastasis staging, infiltration degree or tumor size. The NAG-1 mRNA expression in adenocarcinoma tissues was also lower than that in normal gastric tissues. In conclusion, NAG-1 was poorly expressed in adenocarcinoma tissues and inversely correlated with the degree of tumor differentiation. These results indicate that NAG-1 may have an anti-oncogenic function in the carcinogenesis and development of gastric carcinoma, and that its attenuated or absent expression may lead to gastric carcinogenesis.

## Introduction

There is considerable evidence that nonsteroidal anti-inflammatory drugs (NSAIDs) exert antitumor effects through cyclooxygenase 2 (COX-2)-dependent and independent approaches (1). Furthermore, it has been suggested that nonsteroidal anti-inflammatory drug-activated gene-1 (NAG-1) is capable of inhibiting cell proliferation and promoting apoptosis through various signal transduction pathways. NSAIDs and other chemopreventive phytochemicals are able to induce the expression of NAG-1 in certain tumor cells, and this is considered to be an important non-COX-2 approach by which NSAIDs exert antitumor effects. The role of NAG-1 in gastric cancer carcinogenesis is controversial. We have previously demonstrated that NAG-1 was induced by troglitazone to inhibit the proliferation of a gastric cancer cell line and induce apoptosis *in vitro*(2). It has been suggested that the overexpression of NAG-1 mRNA in invasive areas in gastric tissues functions as a promoter of tumor progression (3). However, in a different study, NAG-1 protein expression was reported to be low in gastric cancer (4). Thus, in the present study, immunohistochemistry and reverse transcription-polymerase chain reaction (RT-PCR) were employed to assess NAG-1 protein and mRNA expression in gastric cancer and normal tissues, with the aim to investigate the possible role of NAG-1 in the carcinogenesis and development of gastric carcinoma.

## Materials and methods

### Research subjects

Forty-six gastric cancer tissue samples were randomly collected from individuals who had undergone gastrectomy for gastric cancer between March 2009 and October 2012 at West China Hospital, Sichuan University (Chengdu, China). The patients included 31 males and 15 females, with a mean age of 56.3±8.1 years. In addition, 26 tumor-adjacent normal tissue samples were collected from 17 male and 9 female patients (mean age, 50.3±9.4 years), and 57 normal gastric mucosa samples were collected by endoscopic biopsy, including 31 males and 26 females (mean age, 57.3±9.97 years). All patients provided informed consent for the biopsy procedure. All paraffin sections were generated and examined using hematoxylin and eosin (H&E) and immunohistochemical staining. Two pathologists independently examined the H&E-stained sections, employing the World Health Organization Histopathological Grading Standards for gastric cancer. Tumor-adjacent normal tissue samples were validated histologically. The fresh tissues were immediately frozen in liquid nitrogen for RNA extraction.

### Antibodies and reagents

Anti-NAG-1 rabbit anti-human polyclonal antibody was purchased from Upstate Biotechnology (Lake Placid, NY, USA), while the SP-9001 immunohistochemistry kit and 3,3′-diaminobenzidine (DAB) were obtained from Zhongshan Biotechnology Co., Ltd., (Beijing, China). Triton X-100 was purchased from Sigma (St. Louis, MO, USA) and TRIzol reagent was purchased from Invitrogen Life Technologies (Carlsbad, CA, USA). A Takara RNA PCR kit (Takara, Shiga, Japan) was used in the study.

### Immunohistochemistry

Placental tissues were used as positive controls. Paraffin sections were deparaffinized and rehydrated and endogenous peroxidase activity was blocked using 3% toluene-hydrogen peroxide. The slides were washed with 0.2% Triton X-100-phosphate-buffered saline (PBS) three times, for 10 min each, and heat-fixed using a pressure cooker with citrate buffer (pH 6.0) for antigen retrieval. A total of 50 μl avidin solution (A solution) and 50 μl D-biotin solution (B solution) was successively added to each slide to further eliminate endogenous avidin biding activity. The slides were incubated with 50 μl rabbit serum for ~20 min and dried, prior to the addition of 50 μl 1:600 NAG-1 polyclonal antibody. Following this, the slides were incubated overnight at 4ºC and washed with PBS three times, for 5 min each time. A total of 50 μl secondary antibody was then added to each slide and incubated at 37ºC for 20 min. The immunoreaction was developed by incubation with streptavidin horseradish avidin and DAB chromogen. The integrated optical density of each slice was assessed using Image-Pro Plus 5.0 Image Analysis Software (MediaCybernetics, Rockville, MD, USA).

### RT-PCR detection

Total RNA was extracted from the fresh tissues using a TRIzol kit, according to the manufacturer’s instructions. The RT-PCR was designed in a two-step method. The primer sequences used in the study were as follows: NAG-1 forward, 5′-GCAAGTGACCATGTGCATCGG-3 and reverse, 5′-CAGGAATCGGGTGTCTCAGGAAC-3′; β-actin forward, 5′-GGGCATGGGTCAGAAGGATT-3′ and reverse, 5′-ATGAGGTAGTCAGTCAGGTC-3′. The cDNA synthesis reaction conditions were as follows: 30ºC for 10 min, 42ºC for 60 min, 99ºC for 5 min and 5ºC for 5 min. The PCR system was utilized according to the manufacturer′s instructions, with the following reaction conditions: Denaturation for 30 sec at 94ºC, annealing for 30 sec at 60ºC, extension for 45 sec at 72ºC, 30 cycles, extension for 10 min at 72ºC and cooling for 10 min at 4ºC. The final PCR products were loaded onto 1% agarose gels and images were captured under ultraviolet light. The objective band and β-actin gray value of the PCR products were measured using Quantity One software^®^ (Bio-Rad, Hercules, CA, USA) and the ratio was taken as an indicator of NAG-1 expression intensity. The PCR products were sent to Shanghai Invitrogen Biotechnology Co, Ltd. (Shanghai, China) for sequencing.

### Statistical analysis

SPSS 13.0 statistical software (SPSS, Inc., Chicago, IL, USA) was used for the analysis. One-way analysis of variance was employed for the comparison between the groups showing normal distribution and the Student-Newman-Keuls method was used for pairwise comparisons. The completely randomized rank sum test was employed for comparisons between two groups of non-normal data. The correlation between tumor-node-metastasis (TNM) staging, infiltration degree, tumor size, differentiation and the expression of NAG-1 was analyzed using the Spearman’s correlation. P<0.05 was considered to indicate a statistically significant difference.

## Results

### Comparison of the NAG-1 protein expression between normal gastric and gastric carcinoma tissues

**Figure 1:**
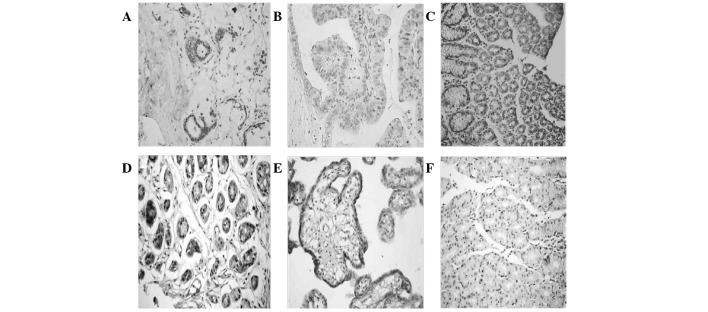
Nonsteroidal anti-inflammatory drug-activated gene-1 protein expression in gastric carcinoma and normal gastric tissues. (A) Poorly differentiated gastric cancer, (B) well-differentiated gastric cancer, (C) tumor-adjacent normal tissues, (D) normal gastric mucosa, (E) placental tissues and (F) normal gastric mucosa (phosphate-buffered saline control) (magnification, ×400).

NAG-1 protein was expressed in the cytoplasm of the placental and normal gastric tissue cells (Fig. 1). Semi-quantitative analysis indicated that the expression of NAG-1 in tumor-adjacent normal gastric tissues was significantly higher than that in the normal gastric mucosa from the endoscopy biopsy (P=0.015; Table I). NAG-1 protein expression levels were lowest in gastric carcinoma tissues and this expression was significantly lower than that in tumor-adjacent normal tissues (P=0.014), as well as lower than that in normal gastric mucosa (P=0.02; Table II).

### Correlation between NAG-1 expression and degree of tumor differentiation, TNM staging, infiltration degree and tumor size

Semi-quantitative immunohistochemical analysis showed that NAG-1 protein expression in moderately and well-differentiated adenocarcinoma tissues was higher than in poorly differentiated adenocarcinoma tissues (P=0.005; Table III). Spearman’s correlation analysis showed that the degree of tumor differentiation and NAG-1 expression intensity were correlated (r=0.854; P=0.03). There was no variations in NAG-1 expression intensity in gastric cancer at different TNM stages (stages I–II and III–IV), infiltration degrees (T0–T2 and T3–T4) or tumor sizes (diameter, ≥5 and <5 cm; Table IV). Spearman’s correlation analysis indicated that there was no correlation between NAG-1 expression intensity and the TNM stage, infiltration degree or tumor size of gastric cancer (r=−0.22, 0.007 and −0.138, respectively).

### Comparison of the expression of NAG-1 mRNA between normal gastric and gastric carcinoma tissues

**Figure 2:**
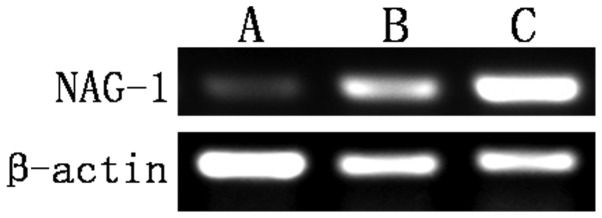
NAG-1 mRNA expression in normal gastric and gastric carcinoma tissues. A, gastric cancer tissues; B, normal gastric mucosa; C, tumor-adjacent normal tissues. NAG-1, nonsteroidal anti-inflammatory drug-activated gene-1.

Gastric carcinoma tissues expressed the lowest levels of NAG-1 mRNA. The expression of NAG-1 mRNA in the tumor-adjacent normal gastric tissues was higher than that in the normal gastric mucosa (Table V and Fig. 2).

## Discussion

NAG-1, a member of the TGF-β superfamily, was originally identified in sulindac sulfide-treated HCT-116 colon cancer cells (5). It was later suggested that a variety of NSAIDs were able to induce NAG-1 gene expression to exert antitumor effects, independent of COX-2. Therefore, this was considered to be one of the most important non-COX-2 approaches by which NSAIDs elicited antitumor effects. In addition to NSAIDs, a number of phytochemicals, including resveratrol (6), genistein (7), diallyl disulfide (8), indole-3-methanol (9), retinoic acid (10) and PPARγ; ligands (11), have been shown to be capable of promoting apoptosis and mediating antitumor effects by inducing the expression of NAG-1. NAG-1, also known as placental transforming growth factor β (12), macrophage inhibitory cytokine 1 (13), placental bone morphogenetic protein (14), prostate differentiation factor (15) and growth differentiation factor 15 (16), is highly expressed in the human placenta and prostate and weakly expressed in the kidney and pancreas (15). The NAG-1 prodomain consists of 167 amino acids and contains an *N*-linked glycosylation site (17). Following dimerization of the full-length pro-NAG-1 precursor by a disulfide linkage, the dimeric pro-protein undergoes proteolytic cleavage catalyzed by furin-like protease at the sequence RXXR, resulting in the release of a 112-amino acid C-terminal dimeric mature region. The mature dimer is then secreted into the extracellular media. Therefore, NAG-1 may have multiple forms in the cell, including the pro-NAG-1 monomer, the pro-NAG-1 dimer, the pro-peptide N-terminal fragment following cleavage and the mature dimer.

The role of NAG-1 in the development and progression of cancer is complex and poorly understood. *In vitro* and *in vivo* studies in colon and prostate cancer and some experimental evidence have suggested that NAG-1 exhibits tumor-suppressor activity (18–21), while other data have suggested that it has oncogenic activity (22,23). Similarly, the role of NAG-1 in gastric cancer carcinogenesis is also controversial. NAG-1 has been demonstrated to stimulate the growth of a number of gastric cell lines, mediated by the activation of the extracellular signal-regulated kinase 1/2 (ERK1/2) pathway (3). In addition, NAG-1 has been shown to activate the protein kinase B and ERK1/2 pathways in human breast and gastric cells by the transactivation of the ErbB2/human epidermal growth factor receptor 2 oncogene (24). A clinical study revealed that NAG-1 expression was upregulated in the serum of patients with gastric cancer and that its expression markedly correlated with cancer metastasis, suggesting an oncogenic role for NAG-1 during gastric cancer progression (25). By contrast, the NAG-1 gene is capable of being induced by NSAIDs (26,27) and troglitazone (2) to inhibit the proliferation of the gastric cancer cell line and induce apoptosis *in vitro*, suggesting that NAG-1 functions as a tumor suppressor in the development of gastric cancer.

In the present study, it was observed that NAG-1 protein expression levels were lowest in gastric carcinoma tissues, and that this expression was significantly lower than that of tumor-adjacent normal tissues, as well as normal gastric mucosa. This suggested that NAG-1 may function as a tumor-suppressor gene in gastric cancer carcinogenesis. The expression of NAG-1 protein in human gastric carcinoma was further analyzed to evaluate its correlation with specific clinical features. NAG-1 protein expression exhibited no correlation with tumor infiltration degree, TNM stage or tumor size, which was inconsistent with the study by Park *et al*(4). The NAG-1 protein expression intensity was inversely correlated with the differentiation of gastric cancer, suggesting that NAG-1 may be involved in regulating the differentiation of gastric cancer. Furthermore, the NAG-1 protein expression in tumor-adjacent normal gastric tissues was higher than that in the normal gastric mucosa, which was attributed to the relatively superficial sampling of the endoscopic biopsy.

NAG-1 expression in normal and cancer tissues has been investigated in a number of studies, which were subsequently reviewed by Mimeault and Batra (28). Collectively, there is no clear consensus regarding the expression levels of NAG-1 in tumors compared with normal tissues, although the majority of the data indicate higher expression in tumors relative to normal tissues. One consideration is the variations in methodologies used to measure NAG-1 expression by different investigators (29). The specificity of the antibodies used to measure the expression of NAG-1 in a number of the studies is frequently not clearly stated. The use of an antibody that detects the monomer form, while poorly reacting with the dimer form, is likely to yield conflicting expression data when compared with the use of an antibody that reacts well with the dimer and poorly with the monomers.

Notably, it was observed in the present study that NAG-1 protein was exclusively expressed in the cytoplasm of gastric glands in the normal gastric mucosa, which was inconsistent with the results of the study by Kim *et al*(30), in which NAG-1 was exclusively expressed in the colonic epithelial membrane lining. This demonstrates that there are secretory NAG-1 protein forms and variations in the activity of the cleaving enzyme which cleave pro-NAG-1 from the RXXR site in different tissues. The activity of the cleaving enzyme is capable of influencing the level of NAG-1 inside the cell, as the cleaved NAG-1 is rapidly secreted. However, the majority of the studies did not examine the activity of the cleaving enzyme when analyzing NAG-1 expression. Thus, NAG-1 expression studies, which are conducted by the measurement of protein expression, must be assessed with caution. Previously, Kadowaki *et al*(31) performed an ELISA in a glioma cell line and normal and glioblastoma tumor samples, revealing that the correlation between the gene copy number and the expression of the pro-NAG-1 in the cells and the concentration of secreted NAG-1, were inconsistent. In specific cells, the majority of NAG-1 was in the secreted form in the media, while in other cells, NAG-1 remained as the pro-NAG-1 inside the cells. Thus, the measurement of gene copy number is a better estimate of NAG-1. Therefore, in the present study, RT-PCR was performed to assess the expression of NAG-1 mRNA in gastric cancer and normal gastric tissues. In addition, PCR products were confirmed by sequencing. The results showed that the expression of NAG-1 mRNA was low in gastric cancer, significantly lower than that of the tumor-adjacent normal tissues and normal gastric mucosa. This was consistent with the immunohistochemical results, which further demonstrated that the absence of NAG-1 is involved in gastric tumorigenesis.

In conclusion, the present study demonstrated that NAG-1 protein and mRNA levels in gastric carcinoma are significantly lower than those in the tumor-adjacent normal tissues and normal gastric mucosa, suggesting that NAG-1 may have a negative regulatory role in gastric cancer by acting as a tumor-suppressor gene. This indicates that low NAG-1 expression may lead to cancer. In-depth studies of NAG-1 are likely to enhance the understanding of the antitumor effect of NSAIDs and also provide a novel target for the prevention and treatment of gastric cancer.

## Figures and Tables

**Table I:** NAG-1 protein expression in tumor-adjacent normal tissues and normal gastric mucosa.

Group	Cases	Mean IOD value	P-value
Tumor-adjacent normal tissues	19	80.09±13.99	0.015
Normal gastric mucosa	33	30.09±15.45	

NAG-1, nonsteroidal anti-inflammatory drug activated gene-1; IOD, integreted optical density.

**Table II:** NAG-1 protein expression in gastric carcinoma tissues and normal gastric mucosa.

Group	Cases	Median IOD value (Quartile)	P-value
Gastric carcinoma tissues	46	2.46 (0–26.77)	0.02
Normal gastric mucosa	33	33.51 (15.25–42.58)	

NAG-1, nonsteroidal anti-inflammatory drug activated gene-1; IOD, integreted optical density.

**Table III:** NAG-1 protein expression in adenocarcinoma tissues.

Group	Cases	Mean IOD value	P-value
Poorly differentiated gastric cancer	28	1.33±1.18	0.005
Moderately differentiated and well-differentiated adenocarcinoma tissues	18	13.78±6.58	

NAG-1, nonsteroidal anti-inflammatory drug activated gene-1; IOD, integreted optical density.

**Table IV:** Correlation between NAG-1 protein expression and TNM stage, infiltration degree and tumor size of gastric cancer.

Characteristic	Cases	Median IOD value (Quartile)	P-value
Infiltration degree			
T0–T2	20	2.46 (0–29.58)	0.96
T3–T4	26	2.45 (1.58–25.52)	
TNM staging			
Stage I–II	16	3.87 (0–32.36)	0.139
Stage III–IV	30	2.95 (1.43–23.18)	
Tumor size, cm			
≥5	24	2.76 (0–17.99)	0.089
<5	22	3.17 (0–31.02)	

NAG-1, nonsteroidal anti-inflammatory drug activated gene-1; TNM, tumor node metastasis; IOD, integreted optical density.

**Table V:** NAG-1 mRNA expression in normal gastric and gastric carcinoma tissues.

Group	Cases	Mean gray scale	P-value
Gastric carcinoma tissues	19	0.8210±0.10173	
Tumor-adjacent normal tissues	26	1.8246±0.14971	0.012^a^
Normal gastric mucosa	24	1.1675±0.08779	0.027^a^; 0.032^b^

a vs. gastric carcinoma tissues;

b vs. tumor-adjacent normal tissues.

NAG-1, nonsteroidal anti-inflammatory drug activated gene-1.
